# Complementary Immunometabolic Effects of Exercise and PPARβ/δ Agonist in the Context of Diet-Induced Weight Loss in Obese Female Mice

**DOI:** 10.3390/ijms20205182

**Published:** 2019-10-19

**Authors:** Sébastien Le Garf, Joseph Murdaca, Isabelle Mothe-Satney, Brigitte Sibille, Gwenaëlle Le Menn, Giulia Chinetti, Jaap G. Neels, Anne-Sophie Rousseau

**Affiliations:** 1 Université Côte d’Azur, INSERM, C3M, CEDEX 3, 06204 Nice, France; sebastien.le-garf@univ-cotedazur.fr (S.L.G.); Joseph.MURDACA@unice.fr (J.M.); Isabelle.Satney@unice.fr (I.M.-S.); Brigitte.Sibille@unice.fr (B.S.); gwenaelle.lemenn@gmail.com (G.L.M.); jaap.neels@unice.fr (J.G.N.); 2 Université Côte d’Azur, INSERM, C3M, CHU, CEDEX 3, 06204 Nice, France; giulia.chinetti@univ-cotedazur.fr

**Keywords:** regulatory T cells, peroxisome proliferator-activated receptor, inflammation, training

## Abstract

Regular aerobic exercise, independently of weight loss, improves metabolic and anti-inflammatory states, and can be regarded as beneficial in counteracting obesity-induced low-grade inflammation. However, it is still unknown how exercise alters immunometabolism in a context of dietary changes. Agonists of the Peroxisome Proliferator Activated-Receptor beta/delta (PPARβ/δ) have been studied this last decade as “exercise-mimetics”, which are potential therapies for metabolic diseases. In this study, we address the question of whether PPARβ/δ agonist treatment would improve the immunometabolic changes induced by exercise in diet-induced obese female mice, having switched from a high fat diet to a normal diet. 24 mice were assigned to groups according to an 8-week exercise training program and/or an 8-week treatment with 3 mg/kg/day of GW0742, a PPARβ/δ agonist. Our results show metabolic changes of peripheral lymphoid tissues with PPARβ/δ agonist (increase in fatty acid oxidation gene expression) or exercise (increase in AMPK activity) and a potentiating effect of the combination of both on the percentage of anti-inflammatory Foxp3+ T cells. Those effects are associated with a decreased visceral adipose tissue mass and skeletal muscle inflammation (TNF-α, Il-6, Il-1β mRNA level), an increase in skeletal muscle oxidative capacities (citrate synthase activity, endurance capacity), and insulin sensitivity. We conclude that a therapeutic approach targeting the PPARβ/δ pathway would improve obesity treatment.

## 1. Introduction

Potentiated by a sedentary lifestyle, inflammation related to obesity is central in the development of type 2 diabetes mellitus (T2DM) [1,2]. Obesity is known to induce a decrease in glucose uptake and lipid metabolism, and a progressive loss of skeletal muscle function. These effects are associated with mitochondrial dysfunction in skeletal muscle and have been shown to correlate with skeletal muscle macrophage infiltration and insulin resistance (IR) [3,4]. Exercise and dietary changes are recommended as non-drug therapies in the first-line strategies for the reduction of obesity [5,6]. These strategies—independently of body weight loss—may have possible impacts on nutrient availability, which could alter the function and migration of immune cells [7]. Metabolism and immunity are tightly integrated in their respective effects: Inflammation alters tissue cell metabolism, and metabolism plays a predominant role in immune cell function [8]. The effects of exercise on immunometabolism are not well defined. Regular aerobic exercise, independently of weight loss, improves metabolic profile and increases anti-inflammatory plasma IL-10 concentrations [5,9,10]. IL-10 production positively correlates with a higher number of circulating regulatory T cell (Tregs) [11]. This effect is potentially clinically relevant, as enhancing the prevalence of Tregs decreases IR in newly diagnosed T2DM patients [12,13]. In contrast to pro-inflammatory Th17 cells, Tregs are not dependent on aerobic glycolysis and de novo fatty acid synthesis [14,15]. Tregs exhibit increased fatty acid oxidation (FAO) compared with conventional T cells [15,16]. The metabolic phenotype and functionality of Tregs are directed by the transcription factor Foxp3, providing to these cells flexibility in fuel choice, in addition to gaining a survival advantage in environments with elevated fatty acid concentrations [17]. The exceptional plasticity of Foxp3+ Tregs is explained, at least partly, by the higher level of AMP-activated protein kinase (AMPK) activity, a key regulator of fuel choice and mitochondrial lipid oxidation not only in skeletal muscle, but also in Tregs [16,17]. 

Fatty acid oxidation represents a promising target for the modulation of the Th17/Tregs balance in favor of Tregs. This opens the way to a possible metabolic reprogramming of immune cells from a pro-inflammatory to an anti-inflammatory phenotype [18], which could reduce the inflammation and tissue lipotoxicity induced by obesity. Among metabolic modulators of fatty acid metabolism, agonists of the Peroxisome Proliferator-Activated Receptor beta/delta (PPARβ/δ) have been studied this last decade as “endurance-exercise mimetics”, susceptible to becoming therapies for metabolic diseases by increasing skeletal muscle FAO and reducing inflammation [19,20]. We have previously shown that activating PPARβ/δ with the GW0742 agonist increases FAO in CD4+ T cells [21]. In mice subjected to an immune challenge such as experimental autoimmune encephalomyelitis [22] or skeletal muscle regeneration [23], PPARβ/δ agonist treatment was shown to improve the Tregs anti-inflammatory response. Moreover, transgenic mice overexpressing PPARβ/δ specifically in T cells are partially protected against high fat diet (HFD)-induced weight gain and exhibit improved insulin sensitivity independently of their decreased weight gain [24]. It is very likely that a therapeutic approach resulting in activating the PPARβ/δ pathway would favor an anti-inflammatory “exercise-like” immune phenotype. These effects may be beneficial in the context of obesity-induced inflammation, but it still remains unclear if targeting PPARβ/δ improves the classical strategy (diet, exercise) for obesity-associated risk reduction. 

Sex differences exist in response to both diet-induced obesity and physical activity for maintenance of weight loss [25,26,27]. Clinical trials have mainly been performed among obese women while paradoxically, experimental studies have been performed with male rodents. Consequently, there is a strong need for experimental animal studies in females to test the efficacy of such treatments. In this study, we address the question of whether immunometabolic changes induced by exercise training, in a context of weight loss, can be modulated by a complementary treatment with a PPARβ/δ agonist. Diet-induced obese (DIO) female mice were subjected to an 8-week normal diet. We investigated in this context the effects of an 8-week PPARβ/δ agonist treatment (GW0742) combined or not with exercise training. Our results suggest a metabolic reprogramming of peripheral lymphoid tissues with exercise training potentiated by PPARβ/δ agonist, and an increase in the percentage of Tregs. This immune change is associated with a decrease in skeletal muscle inflammation, a reduction of visceral adipose tissue mass, and with an increase in oxidative capacities and insulin sensitivity. This led us to conclude that a therapeutic approach targeting the PPARβ/δ pathway would improve obesity treatment.

## 2. Results

### 2.1. GW0742 Treatment in Obese Mice Improves Diet-Induced Weight Loss, Visceral Fat Mass Reduction, and Insulin Sensitivity

**Figure 1:**
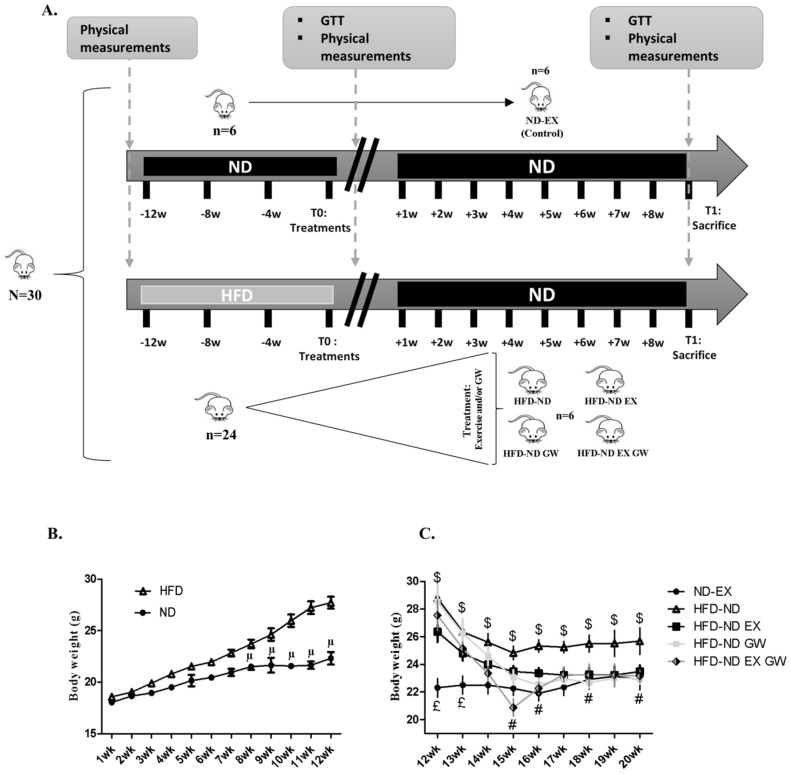
Experimental design of the study. (**A**) 30 mice had free access to a Normal chow Diet (ND) (*n* = 6) or High Fat Diet (HFD) (*n* = 24) for 12 weeks. At T0, they all received a ND for 8 weeks. HFD mice were then randomly assigned in one of four groups: only return to ND (HFD-ND, *n* = 6), return to ND plus exercise training (HFD-ND-EX, *n* = 6), return to ND plus PPARβ/δ agonist GW0742 treatment (HFD-ND-GW, *n* = 6), or return to ND plus combined treatment (HFD-ND-EX-GW, *n* = 6). ND fed mice were maintained on a ND and were trained to be considered as a reference group (ND-EX, *n* = 6). At T0 and T1, glucose tolerance test (GTT) was performed for ND-ex, HFD-ND, and HFD-ND-GW groups, and treadmill endurance test was performed in trained mice (ND-EX, HFD-ND-EX, and HFD-ND-EX-GW). (**B**) Over time representation of weight gain during the 12-week high fat diet (HFD) compared to the normal chow diet (ND). (**C**) Kinetics of weight variation during the 8-wk treatment protocol compared to ND-EX. Data are expressed as mean ± sd.; µ *p* < 0.05 vs. ND; £ *p* < 0.05 vs. all groups; $ *p* < 0.05 vs. ND-EX; # *p* < 0.05 GW0742 effect.

**Figure 2:**
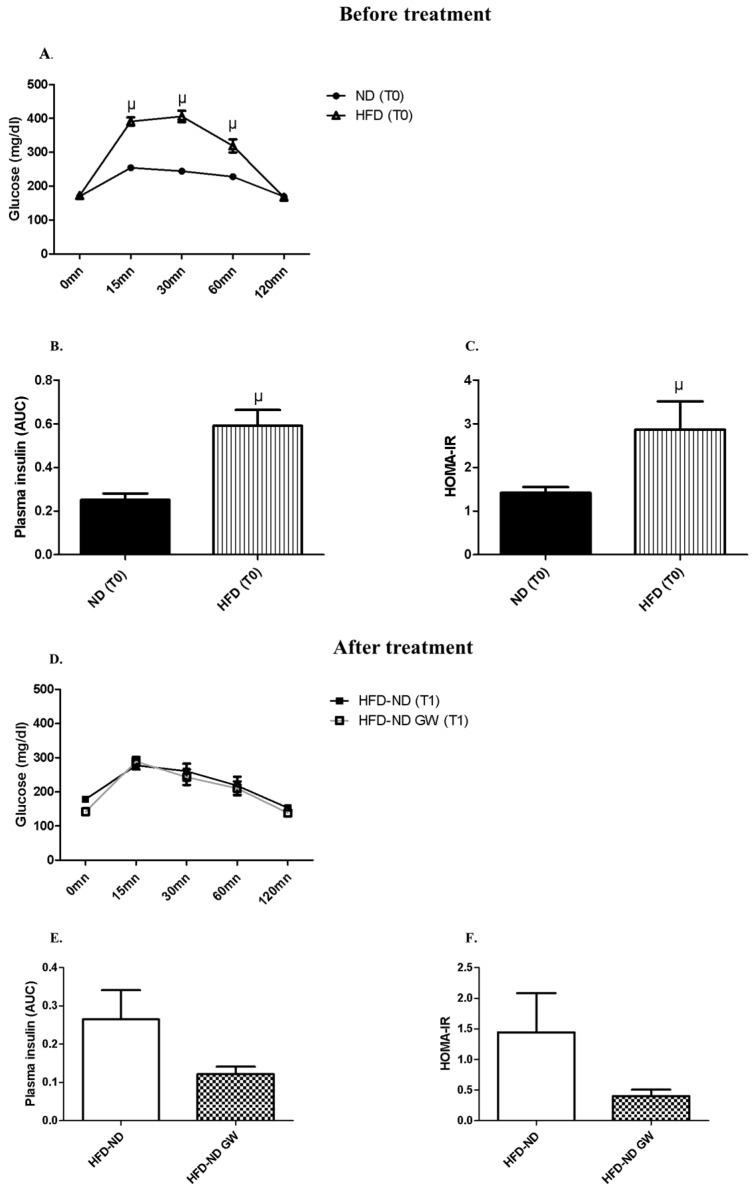
Glucose tolerance curves and insulin plasma concentrations at T0 (after 12-week HFD) and T1 (after 8-week-returning to a ND). (**A**) Glucose tolerance test (GTT) at T0, i.e. after 12-weeks HFD (*n* = 12) or ND (*n* = 6); (**B**) Plasma insulin Area Under the Curve (AUC) during GTT at T0; (**C**) HOMA-IR index calculated with basal blood glucose (mmol/L) and blood insulin during GTT at T0. (**D**) GTT at T1 after returning to ND only (HFD-ND, *n* = 6) or combined with a PPARβ/δ agonist (GW0742) treatment (HFD-ND-GW, *n* = 6). (**E**) Plasma insulin (AUC) during GTT at T1; (**F**) HOMA-IR index at T1. Data are shown as mean ± SD. µ *p* < 0.05 vs. ND at T0.

Twelve weeks of HFD in female mice increased their body weight (Figure 1B), glucose intolerance, and insulin resistance (Figure 2).

Weight reduction following the switch to the ND was significantly higher in GW0742-treated groups, with no significant independent or interaction effect of exercise (Figure 1C and Table 1). Eight-week switching to a ND alone (HFD-ND) did not allow the normalization of visceral and subcutaneous fat masses, which were significantly higher than those of ND-Ex used as “healthy” control mice (Table 1). However, both GW0742- and/or exercise-treated animals had lower adipose masses compared to sedentary mice (HFD-ND group) and even lower than ND-EX (Table 1). No difference was observed for brown adipose tissue mass between all groups (Table 1). Eight-week switching to a ND alone (HFD-ND) did not allow the normalization of those values, which were shown to be significantly higher than those of ND-EX control group (Table 1). At the exception of *vastus lateralis* skeletal muscle, which mass was higher in GW0742-treated exercising animals (HFD-ND-EX-GW), no difference was observed between groups for TLA and soleus mass (Table 1).

As expected, a 12-week HFD led to glucose intolerance and insulin resistance in mice (Figure 2A–C). The switch to ND for 8 weeks restored insulin sensitivity to normal levels (Figure 2). While GW0742 treatment did not affect glycaemia during GTT (Figure 2D) compared to HFD-ND animals (Figure 2D), insulin levels during GTT tended (*p* = 0.08) to be lower (Figure 2E), suggesting that GW0742 treatment led to an improvement in insulin sensitivity. This was confirmed by HOMA-IR, which was lower (*p* = 0.11) in GW0742-treated mice (Figure 2F). Due to technical reasons, we were not able to perform GTT on all groups of animals. As exercise training is now well known to improve insulin sensitivity in normal weight and obese mice [28], we preferred having a sufficient number of mice to perform GTT to explore the effect GW0742 during HFD-ND switching. 

Taken together, our data indicate that in DIO female mice returning to a ND, PPARβ/δ agonist GW0742 treatment improves body composition and insulin sensitivity.

### 2.2. GW0742 Treatment but not Exercise Increases PPARβ/δ Activity in Both Skeletal Muscle and Lymphoid Tissues, Whereas Exercise but not GW0742 Treatment Improves AMPK Regulation in These Tissues

**Figure 3:**
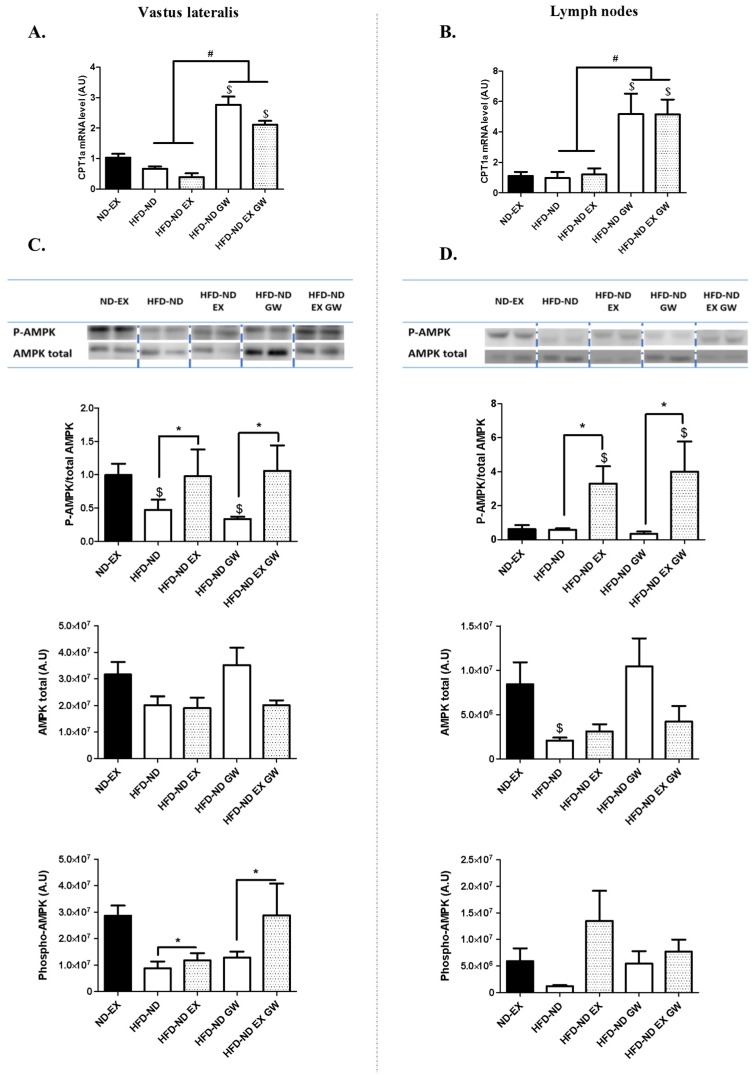
Carnitine palmitoyltransferase 1a (CPT1a) mRNA and AMP-activated protein kinase (AMPK) protein levels in skeletal muscle and lymph nodes. (**A**) CPT1a mRNA level in *vastus lateralis* and (**B**) in lymph nodes. (**C**) and (**D**) Phosphorylated (Thr172) AMPK and total AMPK protein levels measured by western blot in vastus lateralis (**C**) and in lymph nodes (**D**). Data are shown as mean ± SD. (*n* = 6 per group). Blots are shown for *n* = 2 per group. * *p* < 0.05 exercise training effect (2-ways ANOVA); # *p* < 0.05 GW0742 effect (2-way ANOVA); $ *p* < 0.05 vs. ND-EX (one-way ANOVA).

We investigated the expression levels of Carnitine palmitoyltransferase 1a (Cpt1a)—a rate limiting enzyme for mitochondrial fatty acid uptake and β-oxidation—and a previously identified PPARβ/δ target-gene in various tissues and cells including skeletal muscle and CD4+ T cells. The switch to a normal diet alone or combined with exercise had no effect on *Cpt1a* level in these tissues (Figure 3A,B). Eight-weeks GW0742 treatment during the switch to a ND increased by 2-fold Cpt1a mRNA level in the vastus lateralis with no additional effect of exercise (Figure 3A). In lymph nodes—where more than 60% of cells are T cells—*Cpt1a* mRNA level was 5-fold higher in GW0742-treated animals with no additional effect of exercise (Figure 3B). Concerning PPARs isoforms (PPARβ/δ, PPARα, PPARɣ), no effect of GW0742 treatment was evidenced on mRNA levels in both skeletal muscle and lymph nodes (data not shown).

The energy sensor AMP-activated Protein Kinase (AMPK) is one of the key regulators of T cell metabolic adaptation and effector responses in vivo [29,30]. Its activity is shown to be increased in skeletal muscle and in C2C12 cells with short-term treatment with GW0742 [31]. We investigated whether such effects would be observed with long-term GW0742 treatment, and if AMPK expression would also vary in lymphoid tissues. We performed western blot analyses to quantify phosphorylated (P-AMPK Thr172) and total AMPK forms. Exercise but not GW0742 treatment had significantly increased the proportion of P-AMPK to AMPK in both skeletal muscle and lymph nodes (Figure 3C,D). The proportion of P-AMPK to AMPK in skeletal muscle was correlated to the one measured in lymph nodes (*r* = 0.58; *p* = 0.009; not shown). The increase in P-AMPK level in exercising animals was significant in skeletal muscle, but not in lymph nodes (Figure 3C,D). However, the total form of AMPK was not significantly different according the treatment groups in skeletal muscle (Figure 3C), but was lower in HFD-ND animals compared to ND-EX in lymph nodes (Figure 3D). Exercising in HFD-ND mice allowed to restore P-AMPK to AMPK ratio to levels observed in the ND-EX reference group in skeletal muscle. In contrast, sedentary groups had P-AMPK to AMPK ratio significantly lower than those evaluated in the ND-EX reference group. In lymph nodes, the proportion of P-AMPK to AMPK was significantly higher in exercising mice that had been switched from a HFD to a ND compared to ND-EX reference group (Figure 3D). We have next examined the regulation of Acetyl-CoA Carboxylase (ACC), a target protein of AMPK and a potential endogenous indicator of AMPK activity. We performed western blot of ACC phosphorylation (P-ACC) on serine 79 (Figure S1) in skeletal muscle. The proportion of P-ACC to total ACC was improved with the combination of both GW0742 treatment and exercise (interaction effect between exercise and GW0742) (Figure S1). This suggests that the increase in AMPK regulation with exercise in skeletal muscle allowed the increase in its activity in GW0742-treated animals. Due to insufficient quantity of biomaterial, we were not able to performed ACC protein quantification in lymph nodes. Our results show a metabolic reprogramming of peripheral lymphoid and skeletal muscle tissues with PPARβ/δ agonist (increase in fatty acid oxidation gene expression) or exercise (increase in AMPK activity).

### 2.3. Prevalence of CD4+Foxp3+ Regulatory T cells increases with GW0742 Treatment in Lymph Nodes

**Figure 4:**
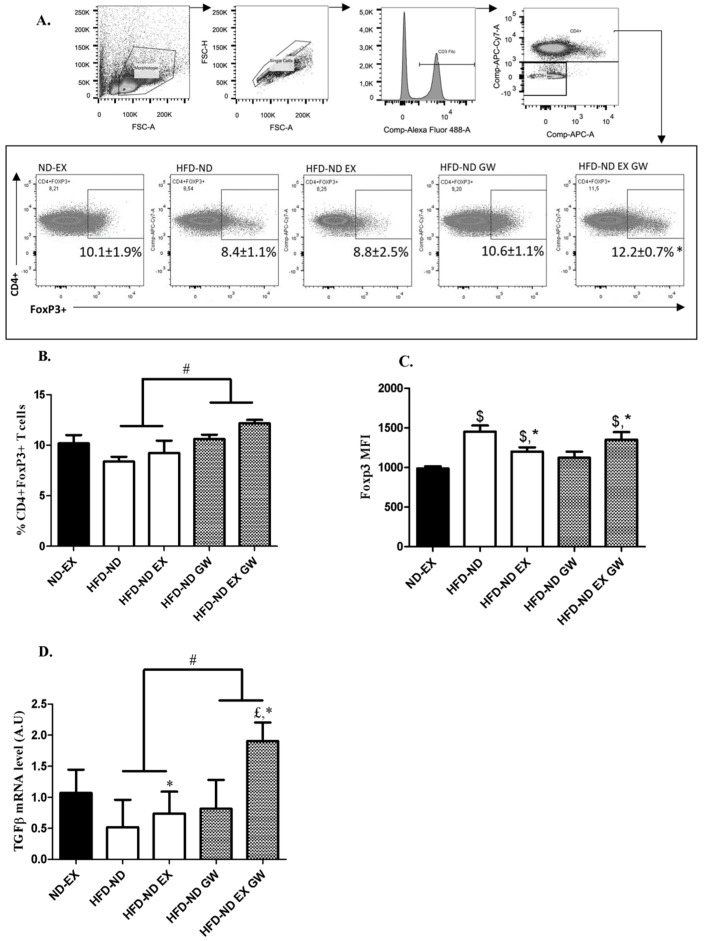
CD4+ expressing Foxp3 T cells in lymph nodes and *TGF-β* mRNA levels. (**A**) Gating strategy of flow cytometry analysis of Foxp3+ on CD4+ cells from lymph nodes. (**B**) Frequency of CD3+CD4+Foxp3+T cells from lymph nodes after return to ND (T1). (**C**) Mean Fluorescence Intensity (MFI) in the APC fluorescence channel (gated Foxp3+ in **A**). (**D**) mRNA levels of *TGF-β* relative to 36B4 in lymph nodes. Data are shown as mean ± SD (*n* = 6 per group). # *p* < 0.05, GW0742 effect and * *p* < 0.05, interaction effect between exercise and GW0742 (2-way ANOVAs). $ *p* < 0.05 vs. ND-EX and £ *p* < 0.05 vs. all groups (one-way ANOVAs).

AMPK activity and FAO are known to be involved in Tregs fate. Tregs display high rates of FAO and an oxidative metabolic profile which is driven in part by Foxp3-dependent expression of genes involved in oxidative metabolism. We investigated by flow cytometry whether exercise and/or GW0742 treatments would alter Foxp3 expression of CD4+ T cells in lymph nodes. We first determined the prevalence of CD3+CD4+Foxp3+ T cells (Figure 4A). No effect of exercise was observed on the prevalence of these cells. However, their percentage was significantly and independently increased in GW0742-treated animals (Figure 4B). When considering the mean fluorescence intensity (MFI)—which characterizes the mean content level of Foxp3 protein in CD4+ T cells—interaction effect between GW0742 treatment and exercise was significant, but independent effect of GW0742 treatment was not (Figure 4C). Except for HFD-ND-GW group, Foxp3 MFI was significantly higher in all HFD groups switching to ND compared to ND-EX reference group (Figure 4C).

As Tregs can control the inappropriate adaptive immune response by producing IL-10 and transforming growth factor beta (TGF-β), we next investigated whether immunoregulatory cytokines would be altered by treatments during the 8-week-shifting to a ND. We performed qPCR analysis on lymph nodes to quantify mRNA levels of *IL-10* and *TGF-β*. Results showed that *TGF-β* mRNA levels were increased with exercise (*p* = 0.0012) and GW0742 treatment (*p* = 0.0004). Moreover, a significant interaction effect between exercise and GW0742 (*p* = 0.03) was observed, with a 2-fold increase in *TGF-β* mRNA levels with the combination of both exercise and GW0742 (Figure 4D). No effect was observed for *IL-10* mRNA levels (data not shown). Although differences exist between exercise and GW0742 treatment, overall these data support adaptations of Tregs after GW0742 treatment (increase in Tregs prevalence and function) with a potential additional effect of exercise.

### 2.4. Exercise Training Reduces Systemic and Skeletal Muscle Inflammation and Impacts Metabolism with Possible Additive Effect of GW0742 Treatment

Enhancing presence of Tregs and limiting the proinflammatory milieu may improve IR in newly diagnosed T2DM patients [12]. We have first performed analysis of plasma inflammatory cytokine concentrations by Luminex to determine whether treatments would impact systemic low-grade inflammation. Analysis showed undetectable pro-inflammatory cytokines in ND-EX reference group. However, IFN-γ, IL-1β, IL-6, and TNF-α levels were detectable in at least half of animals in HFD-ND groups. In contrast, all GW0742-treated and exercising animals had undetectable plasma concentrations of IFN-γ, IL-1β, and TNF-α. Only HFD-ND animals (3 to 6) had detectable concentrations of IFN-γ. The variability of concentrations between mice was high, but this result suggests that 8-week return to a ND alone was not sufficient to blunt systemic inflammation in all mice, whereas addition of GW0742 treatment or exercise seemed to blunt pro-inflammatory cytokine production (Table S1).

**Figure 5:**
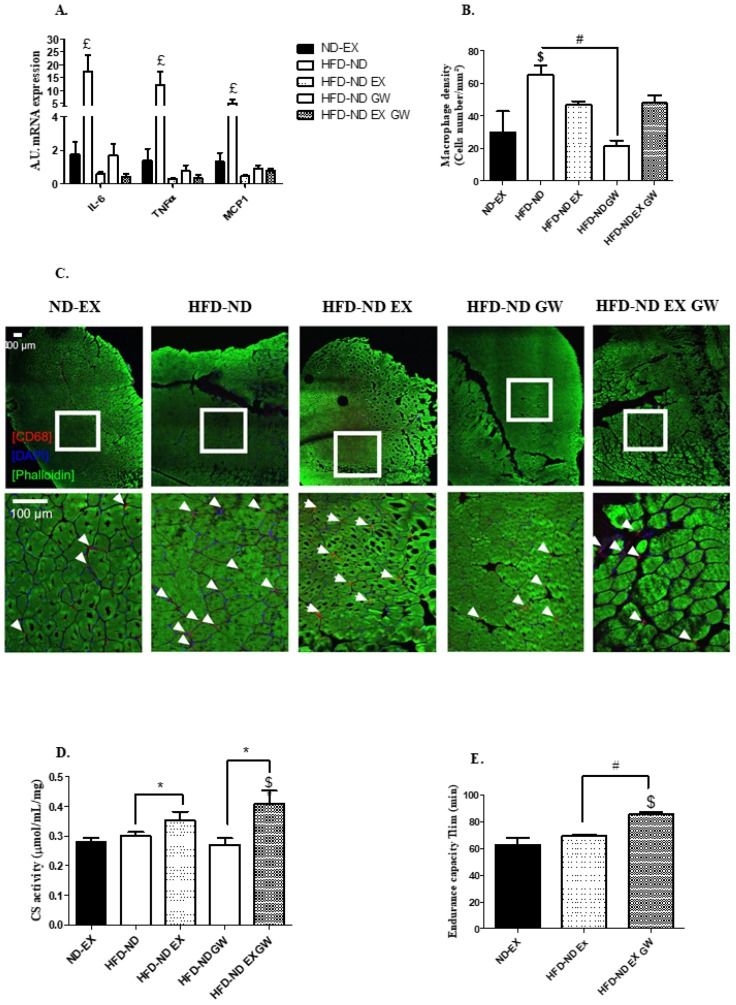
Inflammatory and metabolic states of skeletal muscle. (**A**) Relative mRNA levels of *IL-6*, *TNF-α*, and *MCP-1*. Data are expressed as arbitrary units of expression (A.U.) relatively to *36B4* in the vastus lateralis. (**B**,**C**) Macrophage infiltration by immunohistology staining with Alexa-Fluor488 (staining of actin filament) conjugated phalloidin, CD68 (staining of myeloid cells), and DAPI (staining of cell nucleus) in tibialis lateralis anterior. White borders show the selected area for magnification. White arrows represent infiltrated macrophages. Data are shown as macrophage density (cell number per mm²) for ND-EX (*n* = 3), HFD-ND (*n* = 6), HFD -ND-EX (*n* = 4), HFD-ND-GW (*n* = 6), and HFD-ND-EX-GW groups (*n* = 6). (**D**) Citrate synthase activity is expressed in µmol/mL/mg of vastus lateralis tissue. (**E**) Endurance performance is expressed as the time to exhaustion in minutes of treadmill running (Tlim) for exercise trained groups (ND-EX, HFD-ND-EX, HFD-ND-EX-GW). Data are expressed as mean ± SD. *n* = 6 per group. # *p* < 0.05 GW0742 effect; * *p* < 0.05 exercise training effect (2-way ANOVAs); $ *p* < 0.05 vs. ND-EX; £ *p* < 0.05 vs. all groups (one-way ANOVAs).

HFD feeding has been described to induce inflammation and metabolic dysfunction in skeletal muscle, liver, and adipose tissue stromal vascular fraction. We investigated whether normal diet switching after HFD-induced obesity associated or not with exercise and/or GW0742 treatment would decrease inflammation and improve metabolism in these tissues. We first performed qPCR analysis of genes involved in inflammation and macrophage recruitment. No difference in expression of pro-inflammatory markers in liver and visceral adipose tissue (stromal vascular fraction) was observed. Values reached at the end of treatments were not different from those measured in ND-EX reference group in these two tissues (Figure S2). However, concerning skeletal muscle inflammation, we showed that *IL-6* and *TNF-α* mRNA levels were more than 10-fold and *MCP-1* mRNA levels were more than 4-fold higher in HFD-ND mice compared to their exercising or GW0742-treated counterparts and compared to ND-EX reference group (Figure 5A). When combined with the ND-switching intervention, exercise and GW0742 interventions restored almost completely normal muscle inflammation, but dietary switching alone did not (Figure 5A). We further characterized the effect of exercise and/or GW0742 treatment on skeletal muscle inflammation by performing immunohistochemical staining of CD68, a selective marker for myeloid cells especially macrophages. The macrophage density in tibialis lateralis anterior (TLA) muscle sections in mice that were only subjected to a change of diet (HFD-ND) was significantly higher compared to ND-EX reference group and HFD-ND-GW group (Figure 5B,C).

To further explore the potential association between skeletal muscle inflammation and metabolism in this context, we measured the citrate synthase (CS) activity in vastus lateralis muscle as a witness of muscle oxidative capacity. Exercise training significantly improved CS activity, but no significant independent effect of GW0742 treatment was observed (Figure 5D). However, HFD-ND-EX-GW animals exhibited levels of CS activity significantly exceeding those of ND-EX animals (Figure 5D). We performed treadmill endurance tests in trained animals, and we showed that HFD-ND-EX-GW animals exhibited significantly higher aerobic endurance performances (exhaustion time) compared to their HFD-ND-EX counterparts, meaning that GW0742 treatment during the training of mice had boosted their endurance capacity even beyond that of ND-EX mice (Figure 5E). Importantly, such a long-term treatment with GW0742 in ND fed animals—not previously subjected to a HFD—did not induce such effects (unpublished data). All interventions following the HFD allowed restoring the loss of muscle strength, at least to a level comparable to that of ND-EX mice (data not shown).

## 3. Discussion

PPARβ/δ is known to be a critical integrator of the benefits of exercise in various organs, such as liver, adipose tissue, or skeletal muscle, and has emerged as a novel therapeutic target for metabolic disorders. The immunoregulatory effects of both conventional strategies (exercise, diet)—prescribed as first-line treatment of obesity and T2DM—and alternative pharmacological strategies using PPARβ/δ specific ligands, have been insufficiently studied, particularly in female mice.

Most studies only focus on adipose tissue and liver inflammation to conclude on beneficial effects of dietary or exercise interventions [32,33]. Skeletal muscle is the primary organ for maintaining whole-body glucose homeostasis. Yet intermuscular and perimuscular fat expansions in obesity have been shown to correlate with skeletal muscle T cell and macrophage infiltration and insulin resistance [3]. The return to a low fat diet (ND) in our study is efficient to normalize both glucose tolerance and inflammatory cytokines expression in liver and adipose tissue (Figure S2), as already observed [33,34], but is not efficient to allow a complete recovery of skeletal muscle inflammation. The relevance of this incomplete efficiency of a uniquely dietary-based intervention to completely reverse the inflammation of all tissues is unknown, but should be considered as potentially involved in the worsening effect of weight regain on obesity-induced inflammation and metabolic dysfunctions [35].

Our result contradicts a priori those of some authors that showed a recovery of skeletal muscle inflammatory cytokines to a level of ND animals with a short-term low fat diet-induced weight loss intervention [34]. However, it is noteworthy that we used as reference group mice that were fed with a normal chow diet during the entire study and that were regularly exercised (ND-EX). This decision was made based on data showing that visceral fat mass of sedentary ND fed animals was increased during the 12-week of normal diet, whereas it remained stable in regularly trained mice (Figure S3). Not only a well-balanced diet, but also regular moderate intensity exercise are now admitted as a reference of preventive lifestyle. Unlike ND sedentary animals, ND-EX mice are able to maintain their body composition and their aerobic capacity, suggesting that the physiological state of these mice is closer to the one expected to be reached by the treatment.

In combination with diet-switching, GW0742 treatment allowed a complete recovery of visceral fat mass and improved insulin sensitivity, as shown by the decrease in AUC of plasma insulin during the GTT in GW0742-treated animals (Figure 2C). The improvement of insulin sensitivity may involve mechanisms different from those involved in the decrease in insulin resistance induced by the dietary intervention. Indeed, a role for PPARβ/δ in pancreatic β-cell functions and insulin release was already described [36]. Moreover, a novel ligand-dependent interaction of PPARβ/δ with T-cell protein Tyrosine Phosphatase 45 was recently identified to blunt IL-6 induced IR [37]. Recent studies have shown that dysfunction or decrease in Tregs correlated with IR, and that enhancing the number of Tregs might reduce IR in newly diagnosed T2DM patients [12]. We have not shown a direct link between lymph node Tregs and insulin sensitivity but, taken together, our results would suggest that the increase in Foxp3+CD4+T cells percentage with GW0742 treatment might have contributed to the improvement of mice metabolic parameters. 

The transcription factor Foxp3 is expressed in naturally arising CD4+ Tregs, and is a key regulator for their development [38] and metabolism [39]. There are important differences between the CD4+ T cell subtypes metabolism, in particular the reliance of Tregs on lipid oxidation [16]. Cpt1a is highly expressed in Tregs compared with Th17 [40], and was shown to be up-regulated by 5-fold in lymph nodes of GW0742-treated animals. This reflected an increase in PPARβ/δ transcriptional activity and probably FAO in this tissue. To our knowledge, this is the first report—and a fortiori in this context—of an in vivo effect of PPARβ/δ agonist treatment on increased FAO gene expression in a secondary lymphoid tissue. Since more than 60% of cells in lymph nodes are T cells, we suggest that this induction of Cpt1a could impact metabolism, subtypes, and function of T-cells. Importantly, we did not observe such effects with exercise.

Increasing the function of Tregs is described to be a first-line anti-inflammatory strategy in T2DM prevention [12,41]. Indeed, the percentage of Foxp3+ Tregs and levels of related IL-10 and TGF-β anti-inflammatory cytokines were shown to decrease among newly diagnosed T2DM patients [12]. The combined effects of exercise and GW0742 treatment improved the prevalence of Foxp3+ T cells and *TGF-β* expression in lymph nodes. We were not able to detect any variation of *IL-10* mRNA and plasma concentration levels with exercise and/or GW0742, in contrast to the increase induced by exercise observed among T2DM patients and among immuno-challenged mice treated with GW0742 [22,23]. This suggests that the increase in *TGF-β* expression in our study was perhaps not directly reflecting the function of Tregs. As in secondary lymphoid organs, TGF-β signaling is critical for the development of CD4+Foxp3+ Tregs [42,43], we assume that the increase in *TGF-β* expression with GW0742 and exercise may mostly be an inducer of Tregs differentiation. Although the direct link to each variable is suggestive, regression analysis showed a strong positive correlation between *TGF-β* mRNA level in lymph nodes and the percentage of Foxp3+CD4+ T cells (*r* = 0.59; *p* = 0.0036, not shown). Our data support an overall cellular adaptation of Tregs with GW0742 treatment with a potential additional effect of exercise.

We show that both lymph nodes and skeletal muscle exhibited an increase in the proportion of P-AMPK to total AMPK with exercise training. Expression of the different isoforms of AMPK is tissue restricted, and functional distinction has been reported for the two catalytic α-subunits [44]. We have indifferently analyzed both α1 and α2 subunits and were unable to characterize if regular exercise effects involved distinct mechanisms in skeletal muscle and in lymph nodes tissues. The anti-inflammatory effects of exercise could be partially explained by an activation of AMPK. The later has been shown to drive naive T cells to differentiate toward Tregs both in vitro and in vivo [45]. In skeletal muscle, training, but not diet alone, restored the regulatory capacity of AMPK (proportion of P-AMPK to total AMPK) and endurance performance. Running performances were shown to be improved (by 44%) in sedentary mice treated with AICAR for four weeks. This effect requires PPARβ/δ activity [46]. Moreover, GW0742 increased running performances and aerobic capacities of trained normal weight C57Bl/6J mice [46]. In our study, the effect of PPARβ/δ agonist administration in HFD-ND trained animals goes beyond the expected effects, as the level of performance significantly exceeded by 36% those of ND-EX trained animals. It is possible that GW0742 treatment had improved adaptive exercise training effects by increasing fatty acids released from adipose tissue and increasing the flexibility of skeletal muscle metabolism. In rodents, high fat feeding has been reported to firstly increase lipid availability and skeletal muscle mitochondrial fatty acid oxidation [47,48,49,50,51] and we actually had observed an increase in *Cpt1a* mRNA level with HFD (data not shown). This could be an adaptation of a potential increase in substrate flux to the mitochondria to cope with excess energy in absence of a simultaneous energy demand and to counteract the lipotoxicity [49,50]. The return to a normal diet without concomitant treatments could then be deleterious to maintain these adaptations, since we observed a decrease in *Cpt1a* mRNA levels which might lower FAO.

It is unknown if there is a link between the increase in aerobic performance and capacity with the combined exercise and GW0742 treatments and the concomitant improvement of both the prevalence of Foxp3+CD4+ T cells and the expression of *TGF-β* mRNA. However, it is noteworthy that a study reported a causal link between an elevated prevalence of circulating Tregs and *TGF-β* level and a high VO2peak [52], meaning that aerobic capacity and Tregs are tightly linked. Although together our results suggest beneficial health effects of combined treatments in obese female mice, it remains however uncertain whether the maintenance of GW0742 treatment over a longer period could alter their normal immune function.

To conclude, we characterized for the first time in female mice, independent effects of both exercise and PPARβ/δ agonist treatment, when combined with switching from a high fat to a low fat diet, on immuno-regulatory potential and skeletal muscle inflammation, and a possible compensatory effect of both on immune fate and skeletal muscle endurance capacities. Overall, these data support that both GW0742 and exercise—independently and differently—alter skeletal muscle and lymph node metabolism in favor to a higher FAO potential. GW0742 treatment, but not exercise, increases PPARβ/δ activity in both skeletal muscle and lymphoid tissues, whereas exercise, but not GW0742 treatment, improves AMPK regulatory capacity in these tissues. Altogether, these data suggest that it may be possible to target metabolic pathways and mediators to control Tregs. This could be beneficial in an obesity-induced inflammation and insulin-resistance context. Our data do not support the contention that GW0742 PPARβ/δ agonist is an “exercise mimetic” treatment of obesity. Evidences from our data mostly suggest beneficial complementary effects of PPARβ/δ agonist and exercise treatments in a context of diet-induced weight loss.

## 4. Materials and Methods

### 4.1. Animals and Treatments

Thirty 7-week C57Bl/6J wild-type female mice were purchased from Charles River (Ecully, France). Mice were maintained in a 12-h light, 12-h dark cycle and received food [UAR (Usine d’Alimentation Rationnelle), Villemoisson sur Orge, France] and water ad libitum. After a week of acclimatization, mice were randomly housed 3 per cage. At the age of 8 weeks, 24 mice were fed a 12-weeks diet enriched with fats (HFD, 60% of energy from lipids) (DIO, TestDiet, Laboratory Diet Feed Only, London, UK). The 24 obese/glucose intolerant mice were then randomly assigned to 4 groups according to different treatment administered during 8-weeks (Figure 1A): (1) DIO sedentary mice on a normal chow diet only: HFD-ND group (*n* = 6); (2) DIO sedentary mice on a normal chow diet containing GW0742 (PPARβ/δ ligand agonist) treatment: HFD-ND-GW; (3) DIO exercise training mice on a normal chow diet: HFD-ND-EX; (4) DIO exercise training mice on a normal chow diet containing GW0742 treatment: HFD-ND-EX-GW. We add a reference group of 6 mice that were maintained on a normal chow diet during the entire protocol and subjected to 8-week exercise training (ND-EX). This group was used as a reference in terms of objectives to be achieved and to avoid the confounding effect of chronic inactivity induced by the duration of the protocol. All mice returned to a normal chow diet (standard chow diet (A04)) administered ad libitum supplemented with GW0742 (3 mg/kg BW/day) or with the vehicle (DMSO 1%). Food was reconstituted as described previously [23,53]. Twice a week, the food was refreshed and animals were weighed. Half of the mice concomitantly to the dietary treatment performed an 8-week physical training program on a treadmill (3 sessions per week). To minimize the stress of the procedure, the habituation to exercise on a treadmill began at T0 until the beginning of treatments (5 min per session, at a maximum speed of 10 cm/s). Then, exercise training duration and speed were progressively increased (from 20 min at 20 cm/s (week 1 and 2) to 45 min at a speed of 30 cm/s (week 7 and 8). After the 20-week protocol (T1), animals were deeply anesthetized (intraperitoneal Ketamine/Xylazine, 100/16 mg/kg). Blood samples were obtained by cardiac puncture. The plasma was separated by centrifugation and stored at −80 °C until analyses. Tissues were collected and snap-frozen in liquid nitrogen and stored at −80 °C until use. All experimental procedures were conducted according to French legislation and to the EU Directive 2010/63 for animal experiments, and were approved by the Institutional Ethic Committee for the Use of Laboratory Animals (CIEPAL-AZUR n°447, 2 November 2018).

### 4.2. Glucose Tolerance Test

After 4 h of fasting, mice were injected intraperitoneally with glucose (1 g/kg) for glucose tolerance test (GTT). Plasma glucose was measured at 0, 15, 30, 60, and 120 min after injection with the One-Touch Verio Pro+ glucose-monitoring system (Lifescan, Issy Les Moulineaux, France). Insulinemia was quantified at 0, 15, and 30 min after glucose injection during GTT with an ultra-sensitive rat insulin Elisa kit (Cisbio Bioassays, Codolet, France) HOMA-IR (Homeostatic Model Assessment for Insulin Resistance) index was calculated based on fasting plasma insulin and glucose values at T0 of the GTT using the formula insulin (mU/L) × glucose (mMol/L)/14.1; then values were normalized to 1 for control mice on ND. Mice were sacrificed a week after the GTT was performed, making it less likely that this procedure had an impact on subsequent analyses.

### 4.3. Physical Tests Measurements

Physical tests were performed at the beginning of the treatment (T0) and 2 days before the sacrifice of mice (T8-week) (Figure 1A). The endurance of the mice was evaluated using a treadmill running test (five-lane motorized treadmill, LE8710 M, Bioseb, Vitrolles, France) at 18 m/min, with a slope of 5°, until exhaustion. Exhaustion was considered at the time when the animal was no longer able to run even after 10 mechanical stimuli. To avoid differences between groups due to the familiarization with the procedure, only exercise-trained mice have performed this test (ND-EX, HFD-ND-EX, HFD-ND-EX-GW).

### 4.4. Lymph Node Collection and Cell Preparation

Inguinal, brachial, and cervical lymph nodes were collected and grounded with a mortar before cells were filtered with a 40 µm strainer to obtain a single cell suspension. One part of these cells was directly stained with fluorescence-conjugated antibodies (Miltenyi Biotec, Paris, France) to determine the frequency of Tregs cells. Four million cells were conserved at −80 °C for protein analysis and 4 million for mRNA analysis. 

### 4.5. Flow Cytometry Analysis

Cells were gently washed twice and resuspended in PBS 0.5% FCS plus 2 mM EDTA. Stained cell preparations were analyzed using a BD FACS Canto II flow cytometer (BD Biosciences, Franklin Lakes, NJ, USA). We used Miltenyi Biotec (Paris, France) antibodies: αCD3-FITC, αCD4-APC-vio770, αFoxP3-APC. The extracellular labeling is done initially with αCD3 and αCD4 for 10 min. After 2 washes with PBS 0.5% FCS plus 2mM EDTA, cells are permeabilized and fixed following the manufacturer’s protocol (Miltenyi Biotec Kit). Our gating strategy in order to quantify Tregs in lymph nodes is first by discrimination of cells by size (FSC-A by SSC-A). Then singlets are distinguished using FSC-H by FSC-A. Live cells are gated with Viability dye (Miltenyi Biotec). Following this strategy, we have discriminated CD3+CD4+ versus CD3+CD4-. Finally, at this stage CD4+ FoxP3+ cells are gated as shown in Figure 4A. 

### 4.6. Western Blots Analysis

Total protein lysates from lymph nodes and skeletal muscles of mice were prepared in lysis buffer (20 mM Tris (pH 7.4), 137 mM NaCl, 0.5% Triton X-100, 10% glycerol, 1 mM Na pyrophosphate, 1 mM orthovanadate, 50 mM β-glycerophosphate, 10 mM EDTA, 1 mM EGTA, 1× Complete protease inhibitor cocktail (Roche; 11 873 580 001)), subjected to an electrophoresis and transferred on polyvinylidene difluoride membrane. The following rabbit primary antibodies were used at the same dilution (1:1000) for immunodetection: AMPKα, which detects endogenous levels of total AMPKα (Cell Signaling Technology, Danvers, MA, USA; no. 2532); phospho-AMPKα, which detects both α1- and α2-phosphorylated isoforms of the catalytic subunit (Cell Signaling; Thr172 40H9 no. 2535); ACC, which detects endogenous levels of all isoforms of ACC proteins (Cell signaling; no. 3662); phospho-ACC, which detects endogenous levels of ACC phosphorylated at ser79 (Cell signaling; Ser79 no. 3661). Images were captured using a Syngene Pxi (Ozyme, GeneSys software, Saint-Cyr-l’Ecole, France) and quantified using GeneTools software from GeneSys. When phosphoproteins were analyzed, the membranes were stripped (for 30 min at 50 °C in buffer containing 2-mercaptoethanol 100 mM, SDS 2% (*v*/*v*), and Tris-HCl 62.5 mM), and reprobed with antibodies recognizing the native protein. Luminescent revelation of the secondary antibodies was done using ECL Western Blotting Substrate (ThermoFisher, Dardilly, France).

### 4.7. RNA Extraction and Quantitative Real-Time PCR

Total RNA was extracted with Trizol reagent following the supplier’s protocol (Invitrogen, Dardilly, France). One μg of RNA was reverse-transcribed using a QuantiTect Reverse Transcription Kit (Qiagen) on a Qcycler II (Quanta Biotech, Surrey, UK). Quantitative PCR was performed using SYBR Premix (TAKARA BIO Inc., Shiga, Japan) on a StepOne machine (Life Technologies, Dardilly, France). The relative amount of all mRNAs was calculated using the comparative ∆∆*C*t method, and 36B4 was used as the invariant gene control for all conditions. The mice used for the reference corresponded to the ND-EX group. Primer sequences are available upon request. 

### 4.8. Quantification of Cytokines with Luminex

At the end of the protocol, blood was collected by intracardiac puncture using a 22 G × 1.5 needle and S-monovette syringe coated with EDTA, and then centrifuged to obtain plasma samples. Luminex technology (Mouse High Sensitivity T Cell Magnetic Bead Panel (MHSTCMAG-70K); Merck GmbH, Darm stadt, Germany) was used to measure IFN-γ, IL-1-β, IL-10, and TNF-α in plasma. All quantifications were done according to the protocols provided by the manufacturer.

### 4.9. Immunohistological Staining Protocol

Tibialis anterior (TLA) skeletal muscles were frozen in tissue matrix (OCT: Optimal cutting temperature). Frozen tissues were sectioned using a Cryostat CM350 (Thermo Fisher Scientific, Dardilly, France) and cut to 10 microns. Following slides were blocked for 20 min with 3% BSA and soaked in PBS for 5 min three times. Sections were stained with phalloidin conjugated to Alexa-Fluor488 (1:200; Jackson ImmunoResearch) for actin filament staining, and with CD68 (1:200; BioRad, Marnes-la-Coquette, France) primary antibody (α-rat) combined with a secondary antibody conjugated to Cya3, and DAPI (1:500; Sigma, Lyon, France). CD68 is considered as a selective marker for myeloid cells, especially macrophages. Here, it is used for detection of macrophage infiltration in muscle mice. DAPI was used as nuclear staining. Slides were mounted using Vectashield Mounting Media (Vector Laboratories). Images were acquired using a Nikon Confocal A1R microscope (Amsterdam, Netherlands); objectives: 20X plan Apo l objective (NA0.80-WD 1 mm-Dry), steered by Nis Element software (Nikon, confocal version, Amsterdam, Netherlands), and collecting a stack of images along the Z axis with 2 µm interval between optical sections. Laser excitation wavelengths used are 405, 488, 561 nm. Blue, green, and red emitted fluorescence were collected by 1 PMT and 2 PMT GaAsP detectors (Amsterdam, Netherlands), respectively coupled with 450/50, 525/50, 595/50 nm band pass filters (Amsterdam, Netherlands). Individual images were taken across the entire cross-section and assembled into a composite panoramic image with Nis Element software (Nikon). The amount of infiltrated macrophages was reported on the surface of the histological section and represented as the macrophage density per mm². Data are based on individual animal (ND-EX: *n* = 3; HFD-ND: *n* = 6; HFD-ND-GW: *n* = 6; HFD-ND-EX-GW: *n* = 6).

### 4.10. Citrate Synthase Activity

Citrate synthase catalyzes the reaction between acetyl coenzyme A (acetyl CoA) and oxaloacetic acid to form citric acid and CoA with a thiol group (CoA-SH). Citrate synthase activity was measured in muscle lysates by a colorimetric assay based on the reaction between 5′,5′-DiThiobis 2-NitroBenzoic acid (DTNB, 0.1 mM) and acetyl-CoA (0.43 mM) to form TNB, which exhibits maximum absorbance at 412 nm. The reaction was initiated by adding oxaloacetate (10 mM). The change in absorbance at 412 nm was measured spectrophotometrically at 25 °C. All activities were normalized to mg of total proteins.

### 4.11. Statistical Analysis

The results are presented as means ± standard errors. All data were analyzed using Statview and GraphPad Prism v 5.0 software (San Diego, CA, USA). We performed: (1) Two-way ANOVA analyses to investigate the effect of treatment (GW0742), exercise, and/or interaction effects between treatment and exercise in HFD-ND groups, for each dependent variable under consideration and according to assumptions for statistical analysis (i.e., normal distribution, equal variance), and (2) one way ANOVA or non-parametric Kruskall-Wallis tests to investigate differences considering the ND-EX reference control group. Fisher PLSD and Newman-Keuls post hoc tests analyses were performed for multiple comparisons when statistical significance was reached. Statistical significance was accepted at *p* < 0.05.

## Figures and Tables

**Table 1:** Body weight and composition variations according to obesity-treated groups.

		ND-EX Control		HFD-ND		HFD-ND EX		HFD-ND GW		HFD-ND EX GW	
		Mean	SD	Mean	SD	Mean	SD	Mean	SD	Mean	SD
ΔBW (T1−T0) (g)		1.68	1.19	−2.33	1.08	−2.28	1.42	−6.2 #	2.86	−4.4 #	1.98
Adipose tissues (mg/g)	Subcutaneous	17.29	3.73	21.98 $	6.74	14.61 *	2.43	14.33 *	2.86	14.67 *	4.21
	Visceral	12.55	3.36	21.12 $	7.16	11.41 *	3.33	11.77 *	1.10	10.93 *	3.08
	Brown	3.50	1.17	3.54	0.63	2.98	0.27	3.32	0.42	2.85	0.44
Skeletal muscles (mg/g)	TLA	1.87	0.74	1.45	0.18	1.65	0.08	1.59	0.06	1.56	0.11
	Vastus lateralis	6.88	0.48	6.40	0.37	7.03	0.24	6.83	0.64	7.27 *	0.41
	Soleus	0.28	0.02	0.26	0.02	0.33	0.05	0.33	0.15	0.27	0.05

Mice were fed a Normal chow Diet (ND) (*n* = 6) or High Fat Diet (HFD) (*n* = 24) for 12 weeks. At T0, they all received a ND for 8 weeks. HFD mice were then randomly assigned in one of four groups: only return to ND (HFD-ND, *n* = 6), return to ND plus exercise training (HFD-ND-EX, *n* = 6), return to ND plus PPARβ/δ agonist GW0742 treatment (HFD-ND-GW, *n* = 6), or return to ND plus combined treatment (HFD-ND-EX-GW, *n* = 6). ND fed mice were maintained on a ND and were trained to be considered as a “healthy” reference group (ND-EX, *n* = 6). Data are expressed as mean ± SD. * *p* < 0.05 vs. HFD-ND and # *p* < 0.05 GW0742 effect (2-way ANOVA); $ *p* < 0.05 vs. ND-EX (one-way ANOVA).

## Supplementary Materials

The following are available online at https://www.mdpi.com/1422-0067/20/20/5182/s1.

## Abbreviations

AMPK	AMP-activated protein kinase
BW	Body weight
CD3	Cluster of differentiation 3
CD4	Cluster of differentiation 4
CPT-1	Carnitine palmitoyl transferase-1
CS	Citrate synthase
DIO	Diet-induced obesity
DMSO	Dimethyl sulfoxide
EX	Exercise
FAO	Fatty acid oxidation
FCS	Fœtal calf serum
FoxP3	Forkhead box P3
GTT	Glucose tolerance test
HFD	High fat diet
HOMA-IR	Homeostatic model assessment for insulin resistance
IFNγ	Interferon gamma
IL-6	Interleukin 6
IL-10	Interleukin 10
IR	Insulin resistance
MCP1	Monocyte chemoattractant protein 1
MFI	Mean fluorescence intensity
ND	Normal chow diet
PBS	Phosphate-buffered saline
PDK4	Pyruvate dehydrogenase kinase 4
PPAR	Peroxisome Proliferator-Activated Receptor
T2DM	Type 2 diabetes mellitus
TGF-β	Transforming growth factor beta
TLA	Tibialis lateralis anterior
Th1	T helper 1 cells
Th2	T helper 2 cells
Tregs	Regulatory T cells
Th17	T helper 17 cells
