# Supplementary material for: Complementary Immunometabolic Effects of Exercise and PPARβ/δ Agonist in the Context of Diet-Induced Weight Loss in Obese Female Mice
Source: Int J Mol Sci. 2019 Oct 19;20(20):5182. doi: 10.3390/ijms20205182 (PMC6829333; doi:10.3390/ijms20205182)
Supplement: Supplementary file 1 [file ijms-20-05182-s001.pdf]

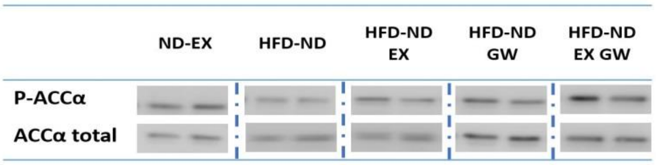

2-way ANOVA: treatment x exercise (p<0.05)

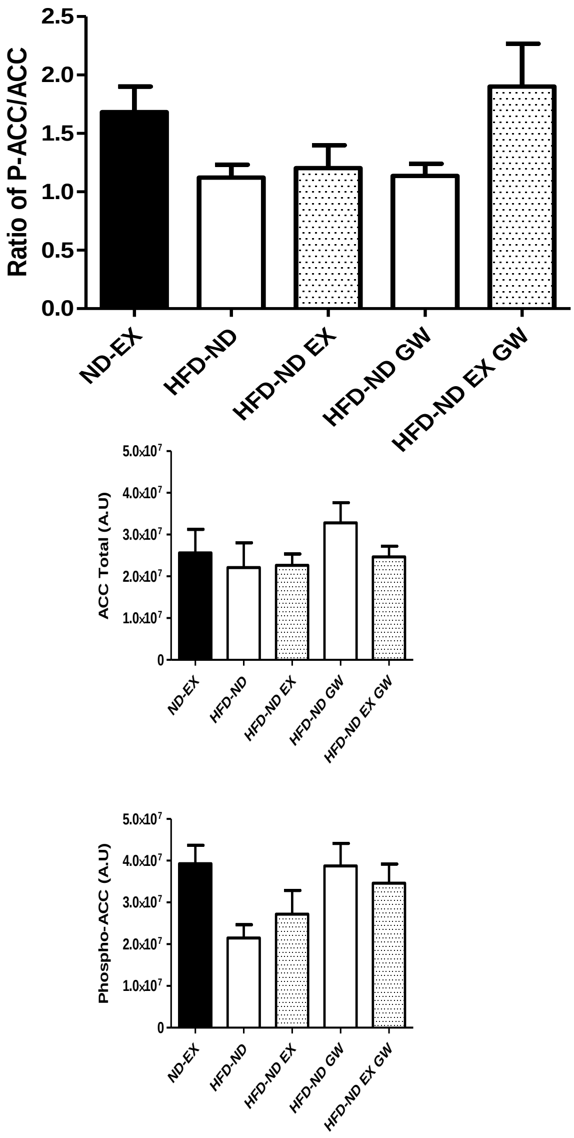

Figure 1. Phosphorylated (Ser79) ACC (P-ACC), total ACC and P-ACC to ACC protein concentrations ratio measured by Western-Blot in *vastus lateralis*.

Data are expressed as mean ± s.d.

A. **Liver**

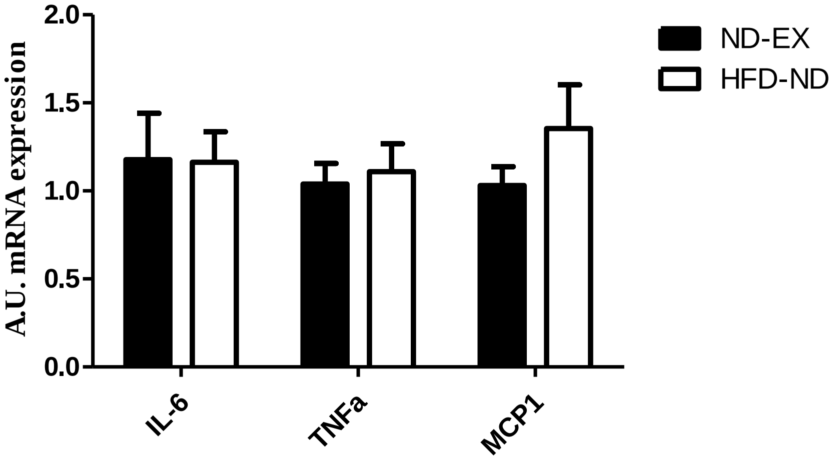

Stromal Vascular Fraction

B.

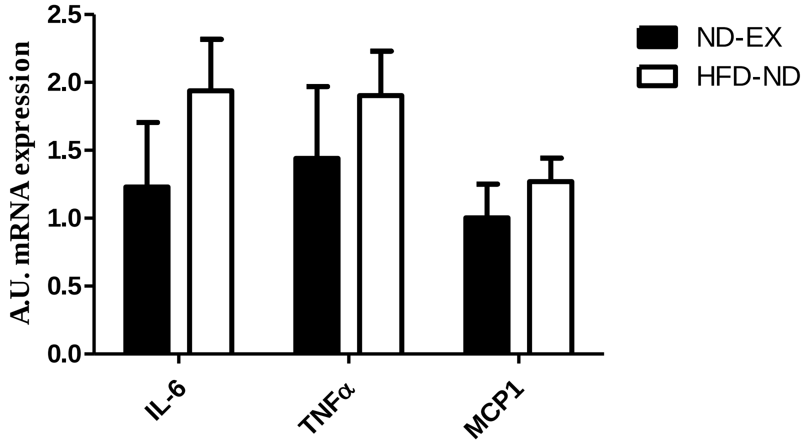

Figure S2: Pro-inflammatory cytokines mRNA levels in liver and stromal vascular fraction.

IL-6, *TNF-α* and *MCP-1* mRNA levels were determined in female mice exposed to a 12-wk HFD and returning to a Normal Diet (HFD-ND) with or without concomitant treatment (HFD-

ND-EX; HFD-ND-GW; HFD-ND-EX-GW). Expression is relative to *36B4*. Data are shown: A) in the liver and B) in the stromal vascular fraction. Data are expressed as mean ± s.d.

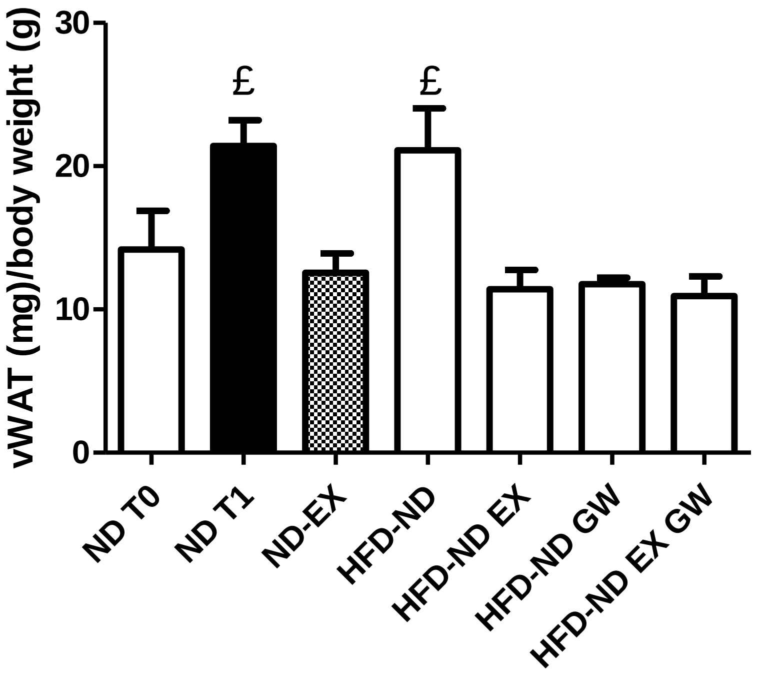

Figure S3: Visceral adipose tissue mass according to treatment groups.

Visceral white adipose tissue (vWAT) mass relative to body weight in all groups before (ND (T0)) and after treatments (ND (T1); ND-EX; HFD-ND; HFD-ND-EX; HFD-ND-GW; HFD-ND-EX-GW). Visceral WAT is expressed in milligram (mg) of tissue per gram of body weight. Data are expressed as mean ± s.d; £ $p<0.05$ vs other groups.

Table S1: Luminex analysis of pro-inflammatory cytokines in plasma.

Results: pg/ml	**IFN-gamma**	**IL1-beta**	**IL-6**	**IL-10**	**TNF-alpha**
**ND-EX**	Und	Und	14.6 ± 9.9	Und	Und
**HFD-ND**	2.67 ± 0.98	22.3 ± 38.8	29.0 ± 48.8	6.11 ± 7.01	7.98 ± 8.68
**HFD-ND EX**	Und	48.0 ± 39.9	70.1 ± 53.6	34.7 ± 35.6	18.9 ± 3.99
**HFD-ND GW**	Und	16.7 ± 6.31	23.0 ± 20.4	5.95 ± 4.63	7.44 ± 2.38
**HFD-ND EX GW**	Und	Und	14.7 ±12.3	4.14 ± 4.7	Und

Und: Undetermined
